# Microvascular Permeability and Texture Analysis of the Skeletal Muscle of Diabetic Rabbits With Critical Limb Ischaemia Based on DCE-MRI

**DOI:** 10.3389/fendo.2022.783163

**Published:** 2022-02-18

**Authors:** Qi Yang, Liang Li, Yunfei Zha, Yuchen Yan, Dong Xing, Huan Liu, Liu Yang, Lin Peng, Yubiao Zhang

**Affiliations:** ^1^ Department of Radiology, Renmin Hospital of Wuhan University, Wuhan, China; ^2^ Advanced Application Team, GE Healthcare, Shanghai, China; ^3^ Department of Orthopedics, Renmin Hospital of Wuhan University, Wuhan, China

**Keywords:** critical limb ischaemia, diabetes, permeability parameter, skeletal muscle, texture analysis

## Abstract

**Background:**

We evaluated skeletal muscle vascular permeability in diabetic rabbits with critical limb ischaemia using quantitative dynamic contrast agent-enhanced (DCE) magnetic resonance imaging (MRI) and explored the feasibility of using DCE-MRI K^trans^-based texture analysis for assessing early slight ischaemia-related skeletal muscle structural changes.

**Method:**

Twenty-four male New Zealand white rabbits (2.7 ± 0.3 kg; n = 12 each in sham-operated and experimental groups) underwent axial MRI of the vastus lateralis muscle at 1, 2, and 3 weeks after alloxan injection. Between-group and intra-group postoperative permeability and texture parameters were compared. Texture features of experimental groups in the third week were modelled by receiver operating characteristic (ROC) curve analysis. Correlations of permeability and of statistical texture parameters with peripheral blood endothelial progenitor cells (EPCs) and microvascular density (MVD) were analysed.

**Results:**

In the experimental group, the transfer constant (K^trans^) was statistically significant at all time-points (*F* = 5.800, *P =* 0.009). Their vastus lateralis muscle K^trans^ was significantly lower in the third than in the first week (*P* = 0.018) and correlated positively with peripheral blood EPCs in the experimental group [*r* = 0.598, (95% CI: 0.256, 0.807)]. The rate constant was negatively associated with vastus lateralis muscle MVD [*r* = -0.410, (95% CI: -0.698, -0.008)]. The area under the ROC curve of texture parameters based on K^trans^ in ischaemic limbs was 0.882.

**Conclusions:**

Quantitative DCE-MRI parameters could evaluate microvascular permeability of ischaemic limb skeletal muscle, and texture analysis based on DCE-MRI K^trans^ allowed evaluation of early slight skeletal muscle structural changes.

## Introduction

Patients with diabetes have a higher risk of developing peripheral artery disease. Percutaneous transluminal angioplasty and surgical bypass are less effective in patients with than in those without diabetes, and perioperative morbidity and mortality are increased and the amputation rates are high in those with diabetes (1). Critical limb ischaemia (CLI) is the latest stage of peripheral arterial disease in patients with diabetes, in which the blood supply to the skeletal muscles of the lower extremities is inadequate, resulting in atrophy and necrosis. Patients are thus at a high risk of amputation or death (2). Therefore, accurate assessment of skeletal muscle microcirculation changes is significant for diagnosis and treatment planning, detection of efficacy, and prognosis evaluation. Although the detailed roles of cytokines and inflammatory cells are unclear, the long-term blood glucose increase, insulin resistance, and other factors cause distortion of the capillaries of the skeletal muscle and swelling of the endothelial cells, resulting in endothelial cell dysfunction and capillary basement membrane thickening, vascular permeability, and changes to the skeletal muscle microstructure. Thus, it is necessary to explore early abnormal changes in the skeletal muscle, predict early unnatural changes, monitor the progress of those changes, and assess the curative effects of treatment in patients with CLI.

Texture analysis (TA) is an image post-processing technique used to study the heterogeneity of lesion tissues through pixel intensity and/or greyscale on magnetic resonance imaging (MRI) and computed tomography (CT), which can be quantified as image heterogeneity caused by changes that cannot be observed by human eyes (3). Tian et al. (4) proved that the transfer constant (K^trans^) of dynamic contrast agent-enhanced (DCE)-MRI can be used as quantify changes in microvascular permeability of the soleus and anterior tibial muscles in lower extremity vascular lesions in the diabetic group. Qi et al. (5)showed that DCE-MRI K^trans^ can be used for early and sensitive diagnosis of denervated skeletal muscle.

However, to date, no study has described a microvascular permeability and texture analysis of the skeletal muscles of diabetic rabbits with CLI using DCE-MRI. In this study, we investigated the longitudinal and horizontal relationship between skeletal muscle vascular permeability and texture in a rabbit model of diabetes with CLI using quantitative DCE-MRI.

## Materials and Methods

Our study began in December 2018 and ended in January 2019. All experiments were conducted with the approval of the Animal Experiment Center at Renmin Hospital of Wuhan University and the ethics committee at Wuhan University. The care of laboratory animals and all animal experiments adhered to the Guide for the Care and Use of Laboratory Animals published by the US National Institutes of Health.

### Model Building of Diabetic Rabbits With Critical Limb Ischaemia

Twenty-four 3-month-old male New Zealand white rabbits were purchased from the Wuhan University Center for Animal Experiments. All rabbits were weighed (mean ± SE = 2.7 ± 0.3 kg). Fasting blood glucose was measured in 24 rabbits after adaptive feeding for 1 week; their blood glucose levels were normal and without significant differences (5.5 ± 0.5 mmol/L).

Rabbits were randomly distributed into an experimental group (n = 12) and a sham-operated group (n = 12). They were fasted for 8 h. A 5% solution of alloxan (Sigma, St Louis, MO, USA) was prepared in normal saline. Rabbits in the experimental group were administered 100 mg of alloxan per kg of body weight by ear vein, within 30 s, to induce diabetes, and the sham-operated group was administered saline without alloxan. Peripheral blood glucose was monitored using a glucose meter(Abbott, Bedford, Mass) every hour for 24 h. A diabetic state was achieved 48 h after alloxan injection, as checked out by mensurable detection of blood glucose levels. In the fasting state, blood glucose levels higher than 14 mmol/L or two measurements higher than 11 mmol/L were deemed to indicate successful diabetes model generation (6).

Two weeks later, all rabbits were anaesthetised by intravenous injection of 30 g/L pentobarbital sodium (1 ml/kg) through the ear margin. After completion of anaesthesia, an oblique incision of approximately 3 cm was made in the right groin in the experimental group in the supine position. The femoral artery was carefully dissociated and separated from the parallel veins and nerves and was ligated at its beginning (inguinal ligament). The sham group repeat the same operation without ligated. The two groups was placed in a supine position in the SONIALVISION G4 in the digital X-ray fluorography system (Shimadzu Corp, Kyoto, Japan). A heparin-soaked 3-F microcatheter was inserted through the common iliac artery, the tip was placed in the common iliac artery, and 20 mL of iodoverol (320 mgI/ml, Jiangsu Hengri Pharmaceutical, Co. Ltd., Lianyungang, China) was manually injected at a flow rate of 1.5 ml/s.

Continuous cineangiography results were also obtained. The imaging speed was 8 frames/s, and the magnification rate was 1.5 ×. The incision was sutured after angiography, and penicillin/streptomycin was used as anti-infection treatment for 3 days after surgery.

### MRI Equipment and Imaging Techniques

At 1, 2, and 3 weeks after successful CLI model establishment, all rabbits were placed in the supine position and fixed in the 8-channel dedicated phase-controlled knee coil of a 3.0-T MR scanner (Discovery MR750 Plus, GE Healthcare, Chicago, IL, USA), which was used to image the right thigh vastus lateralis muscle, using conventional axial DCE-MRI,fast spin echo (FSE)-T1-weighted imaging (WI), and FSE-T2WI.

Axial FSE-T1WI scanning parameters were as follows: TR, 400 ms; TE, 9.6 ms; scanning layer thickness, 3 mm; field-of-vision (FOV), 12 × 12 cm; matrix, 256 × 256; number of excitations, 4. Axial FSE-T2WI scanning parameters were as follows: TR, 2500 ms; TE, 121.7 ms; scanning layer thickness, 3 mm; field of vision, 12 × 12 cm; matrix, 256 ×2 56; excitation, 4.

DCE-MRI uses the liver acquisition volume acceleration (LAVA) sequence and the spatial sensitivity encoding technique (ASSET). First, the LAVA sequence was used in scanning with multiple turn-overs (TR, 3.5 ms; TE, 1.6 ms; thickness, 3.0 mm; FOV, 20 cm × 16 cm; matrix, 192 × 192; and turning angle, 9° and 12°). One time-phase (8 s) was scanned for each multiturn sequence. The LAVA sequence scanning was dynamically enhanced (TR, 3.5 ms; TE, 1.6 ms; layer thickness, 3.0 mm; FOV, 20 cm × 16 cm; matrix, 192 × 192; turning angle, 10°). By continuously scanning 420 frames of dynamic images in the same layer without interval, a total of 35 time-phases were scanned over a period of 4 min 31 s. After baseline scanning of two dynamic time-phases, a dual-cylinder high-pressure syringe (Medrad Spectris Solaris EP Mobile Mount MR Injection System, Leverkusen, Germany) was used to inject gadodiamide (gadolinium diamine, GE Healthcare) into the rabbit auricular veins. The injection dose was 0.2 mmol/kg, and the flow rate was 1.0 ml/s. This was followed by a 0.9% normal saline flush at the same flow rate.

### DCE-MRI Quantitative Parameter Analysis

**Figure 1:**
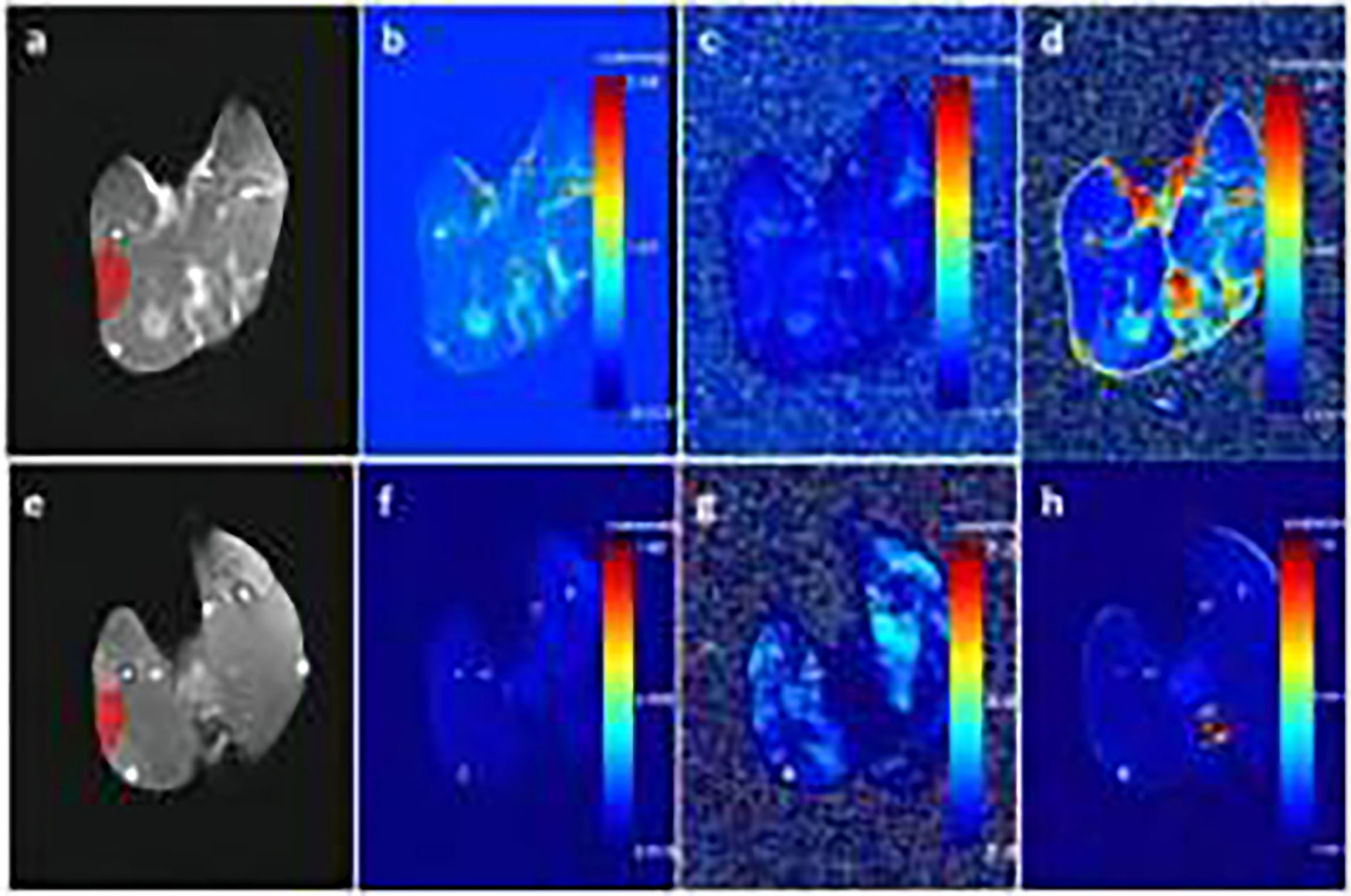
**(A–D)** show enhanced T1-weighted imaging (T1WI), K^trans^, Kep, and Ve of the experimental group in the third week, respectively. **(E–H)** show enhanced T1WI, K^trans^, Kep, and Ve of the sham-operated group in the third week, respectively.

DCE-MRI raw data were imported into Omni-Kinetics (GE Healthcare) software for analysis. First, 3D non-rigid motion correction was performed on the 35-phase dynamic enhanced image to reduce respiratory motion artefacts. Then, the LAVA sequence images with two rotation angles (9° and 12°) were imported for T1 mapping calculation. Next, we imported the modified 35-stage enhanced image and fit the time-concentration curve of the contrast solution inside the aorta as an arterial input function (AIF) of the thigh vastus lateralis muscle, with selection of extended Tofts for the pharmacokinetic model. We selected the midlevel image of the femur and manually drew the region-of-interest (ROI). The ROI was placed so as to avoid the subcutaneous fat layer. Fascia software automatically calculated K^trans^, the rate constant (Kep), and the volume of extravascular extracellular space (Ve) three times for each ROI; these three measurements were then averaged for use (**Figure 1**). **Figures 1A–D** showed the enhanced T1WI, K^trans^, Kep and Ve of the experimental group in the third week, respectively; e, f, g and h showed the enhanced T1WI, K^trans^, Kep and Ve of the sham-operated group in the third week, respectively.

### Image Texture Extraction

Omni-kinetics software (GE Healthcare) was used to select images of the middle segment of the femur in the experimental and sham-operated groups at week 3. The ROI was manually drawn based on the conventional DCE-MRI and the texture feature parameters for the selected ROI were calculated. The ROI encompassed as much of the entire vastus lateralis muscle as possible. A total of 63 texture characteristic parameters were extracted for each selected ROI using omni-kinetics texture analysis software. These parameters included the histograms, morphological,grey-level co-occurrence matrix (GLCM) and grey-level run-length matrix (GLRLM), among others.

### Histopathological Examination

After completion of the MRI examination, all rabbits were sacrificed by air embolisation, and the lateral thigh muscles of the middle femur were removed and fixed in 10% formalin for 24 h. The vertical muscle fibres were cut into 4-μm-thick slices and immunohistochemically stained for CD31. Slices were observed and photographed under an optical microscope (BX51, Olympus, Tokyo, Japan). Image Pro-Plus software (version 6.0) was used for image analysis. Three areas of CD31-immunohistochemically stained sections using the same area of 200× magnification were photographed under the microscope. The capillary density was calculated according to the number of microvessels in these areas. Any brownish-yellow endothelial cell or cell mass isolated from adjacent microvessels and other connective tissue was considered as a single microvessel.

### Peripheral Blood EPCs Count Detection

A 0.5% crystal violet solution was prepared by dissolving crystal violet in methanol, which was then diluted in PBS to a 0.1% solution. Blood was drawn through the auricular vein, Endothelial progenitor cells (EPCs) in each group were digested with trypsin to produce a cell suspension, the suspension was spread evenly on a slide. Wash the cells twice with PBS and resuspend the cells with 100ul PBS. CD31 primary antibodies were added, Sterile Eppendorf tubes were prepared with a 200-μl serum-free medium containing 2.5 × 10^4^ cell suspensions. Cells were inoculated into the upper chamber of a Transwell plate. Complete medium containing 10% fetal bovine serum in 500 µL was added to the Transwell plate, and cells were cultured in a CO_2_ (5%) incubator at 37°C for 24 h. The small chamber was then removed, and the medium was washed out with PBS. Crystal violet staining was performed for 10 min. Excess crystal violet was washed from the surface under tap water. The cells in the upper chamber were then wiped off with a cotton swab, and the non-cell inoculation side was photographed under an inverted microscope. Under the microscope, three visual fields under 200× magnification were selected for counting, and the average value was taken.

### Statistical Analyses

All statistical analyses were implemented by using SPSS 22.0 Windows Student Version statistics software (IBM, Armonk, NY, USA). K^trans^, Ve, Kep, and pathological parameters were tested for normality and homogeneity of variance. Parameters with a standard normal distribution and homoscedasticity are evinced as mean ± standard deviation. Those that did not conform to a normal distribution are shown as the median (upper and lower quartile). Differences in K^trans^, Kep, and Ve in the lateral femur muscles between the diabetic group and the sham-operated group at the same time-point were compared using an independent sample *t*-test (for data with a standard normal distribution and homoscedasticity) or the Mann−Whitney U test (for data without a normal distribution or homogeneity of variance). Repeated-measurements analyses of variance were used to compare differences in K^trans^, Ve, and Kep values at different time-points.

The texture features extracted at the third week in the experimental group and the sham-operated group were modelled by logistic regression analysis after dimensionality reduction. Features were analysed using Mann−Whitney U tests, t-tests, and correlation analysis. Finally, the area under the receiver operating characteristic (ROC) curve was computed to evaluate the diagnostic efficacy of the valuable K^trans^ texture parameters. The difference in MVD (CD31) and peripheral blood EPC count between the experimental and sham-operated groups was compared using a Mann-Whitney U test or independent sample t-test.

Pearson correlation analysis was used to evaluate the correlation betwixt K^trans^, Ve, and Kep osmotic parameters and significant texture parameters with MVD (CD31) and peripheral blood EPCs. All results were reckoned statistically significant at P < 0.05.

## Results

**Figure 2:**
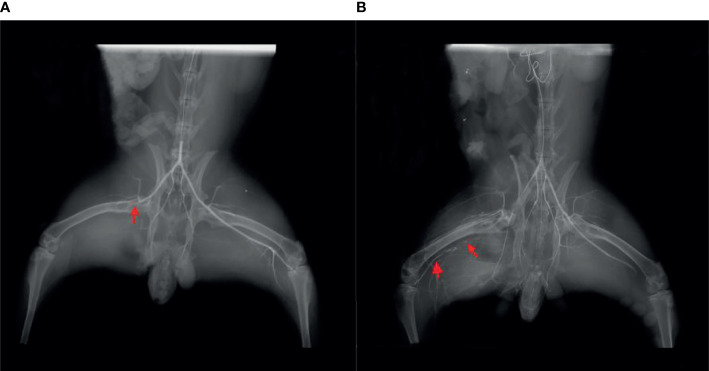
**(A)** Angiography immediately after ligation of the unilateral femoral artery of the lower extremity in rabbits. The thin arrow indicates ligation without recanalization of the femoral artery. **(B)** Angiography of unilateral femoral artery ligation in the lower extremity in rabbits at the third week. The thin arrow indicates ligation without recanalization of femoral artery, and the bold arrow shows the formation of collateral circulation.

Femoral angiography was performed immediately after unilateral femoral artery ligation in rabbits, and this was compared to angiography performed 3 weeks later. Angiography showed circulation indicating occlusion of the distal femoral artery (**Figure 2**), reflecting appropriate establishment of a CLI model. **Figure 2A** showed angiography after ligation of unilateral femoral artery of lower extremity in rabbits immediately, and the thin arrow indicated ligation without recanalization of femoral artery. **Figure 2B** showed the angiography of unilateral femoral artery ligation in lower extremity in rabbits at the 3rd week, and the thin arrow indicated ligation without recanalization of femoral artery, the bold arrow shows the formation of collateral circulation.

K^trans^ in the experimental group showed statistically significant differences between each of the 3 time-points (F = 5.800, P < 0.009), while in the sham-operated group, there was no statistically significant difference (F = 0.533, P > 0.05). K^trans^ in the lateral femoral muscle of the experimental group was decreased in the third week compared with that in the first week (P = 0.018), while pairwise comparison at other time-points showed no statistical significance (P > 0.05). Ve and Kep showed no significant difference between the experimental group and the sham-operated group, at each time-point (F values were 0.991, 0.497, 0.620, 0.671, all P > 0.05) (**Table 1**).

**Table 1:** Comparison of K^trans^, Ve, and Kep at each time-point between the experimental group and the sham-operated group.

Time-point	Experimental group	sham-operated group	*T* (*Z*) value	*P* value
K^trans^(min^-1^)				
1 week	0.112 (0.043, 0.291)	0.119 (0.064, 0.159)	-0.115^a^	0.932
2 weeks	0.103 ± 0.041	0.195 ± 0.139	2.199	0.020^#^
3 weeks	0.087 (0.045, 0.091)^*^	0.166 (0.086, 0.208)	-2.512^a^	0.010^#^
Ve				
1 week	0.146 (0.050, 0.652)	0.098 (0.056, 0.395)	-0.231^a^	0.843
2 weeks	0.037 ± 0.044	0.124 ± 0.050	4.540	0.341
3 weeks	0.130 (0.059, 0.164)	0.081 (0.064, 0.230)	0.404^a^	0.713
Kep (min^-1^)				
1 week	0.706 (0.442, 1.184)	0.802 (0.705, 1.734)	-1.386^a^	0.178
2 weeks	0.680 (0.354, 1.084)	1.237 (0.953, 2.198)	-2.309^a^	0.02^#^
3 weeks	1.091 (0.710, 1.254)	1.463 (0.856, 2.229)	-1.617^a^	0.114

Normally distributed data are expressed as mean ± standard deviation. Non-normally distributed data are presented as median (upper and lower quartiles). ^a^Z value; *a statistically significant difference in K^trans^ between the representative group and week 1 (P < 0.05); ^#^a statistically significant difference in K^trans^ and Kep between the experimental group and the sham-operated group at the same time-point (P < 0.05).

**Figure 3:**
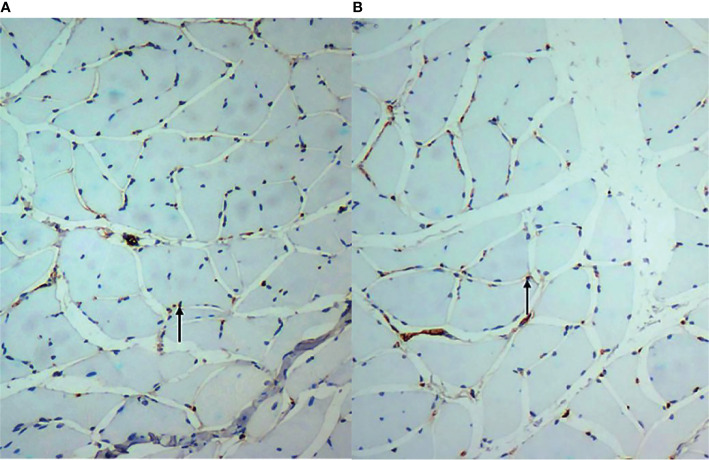
**(A, B)** show CD31 immunohistochemical staining (×200) in the experimental group and the sham-operated group at week 3, respectively. Microvascular density in the experimental group increased compared with that in the sham-operated group. The brown-yellow particles indicated by the black arrows in are CD31-labelled microvessels.

At week 3, CD31 of the vastus lateralis muscle of the experimental group showed an increase in MVD between muscle fibres compared to the sham-operated group (**Figure 3**). **Figures 3A, B** showed CD31 immunohistochemical staining (×200) in the experimental group and the sham-operated group at week 3, respectively. MVD in the experimental group increased compared with that in the sham-operated group. The brown-yellow particles indicated by the black arrows in **Figures 3A, B** are cd31-labeled microvessels.The MVD average values of the experimental and sham-operated groups were 216.100 ± 74.248/mm^2^ and 46.634 ± 32.119/mm^2^, respectively (t = -7.257, P = 0.032).

**Figure 4:**
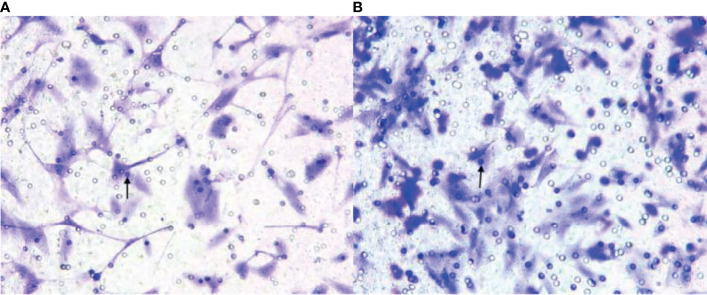
**(A, B)** show endothelial progenitor cells (EPCs) in the experimental group and the sham-operated group at week 3 (×200), respectively. The EPCs in the experimental group were lower than those in the sham-operated group. The black arrows indicate the EPCs migrating to peripheral blood.

The EPC count in the experimental group was lower than that in the sham-operated group (**Figure 4**). **Figures 4A, B** showed the EPCs count in the experimental group and the sham-operated group at week 3 (×200), respectively. The EPCs in the experimental group were lower than those in the sham-operated group. The black arrows in **Figures 4A, B** indicate the EPCs migrating to peripheral blood. The median was 38.000 (34.500, 40.750)/mm^2^ and 49.000 (38.500,49.000)/mm^2^, respectively. Thus, the EPC value of the experimental group was lower than that of the sham-operated group (Z = -2.228, P < 0.05).

**Figure 5:**
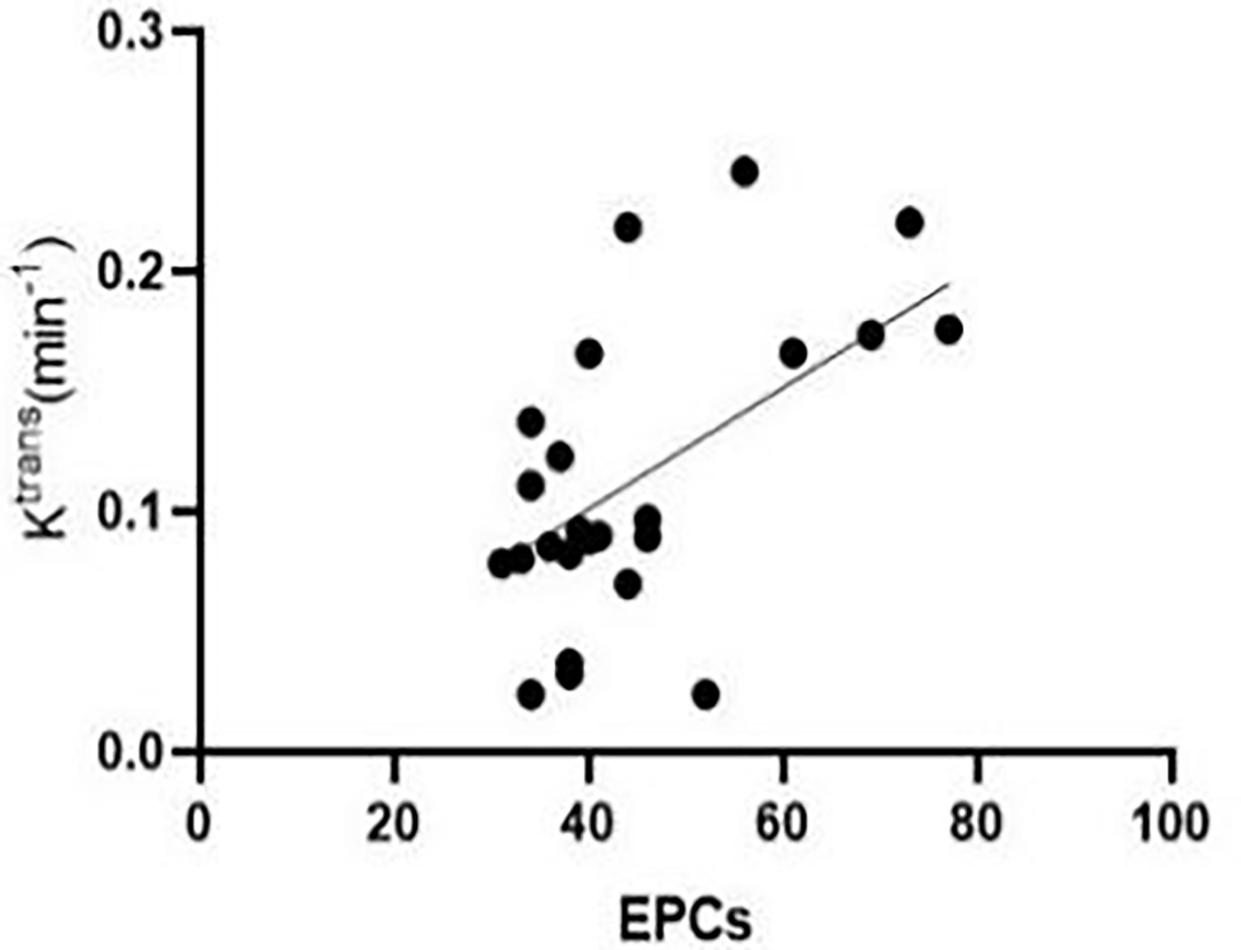
At the third week after successful modelling, EPCs in the peripheral blood of experimental rabbits correlated positively with K^trans^.

At week 3 after successful modelling of CLI, Pearson’s correlation analysis results showed that the number of peripheral blood EPCs in experimental rabbits was positively correlated with K^trans^ [r = 0.598, (95% confidence interval (CI): 0.256, 0.807)] (**Figure 5**). **Figure 5** at the 3rd week after successful modeling, EPCs in peripheral blood of experimental rabbits positively correlated with Ktrans. There was no correlation between MVD and K^trans^ [r = -0.319, (95% CI: -0.640, 0.097)].

**Figure 6:**
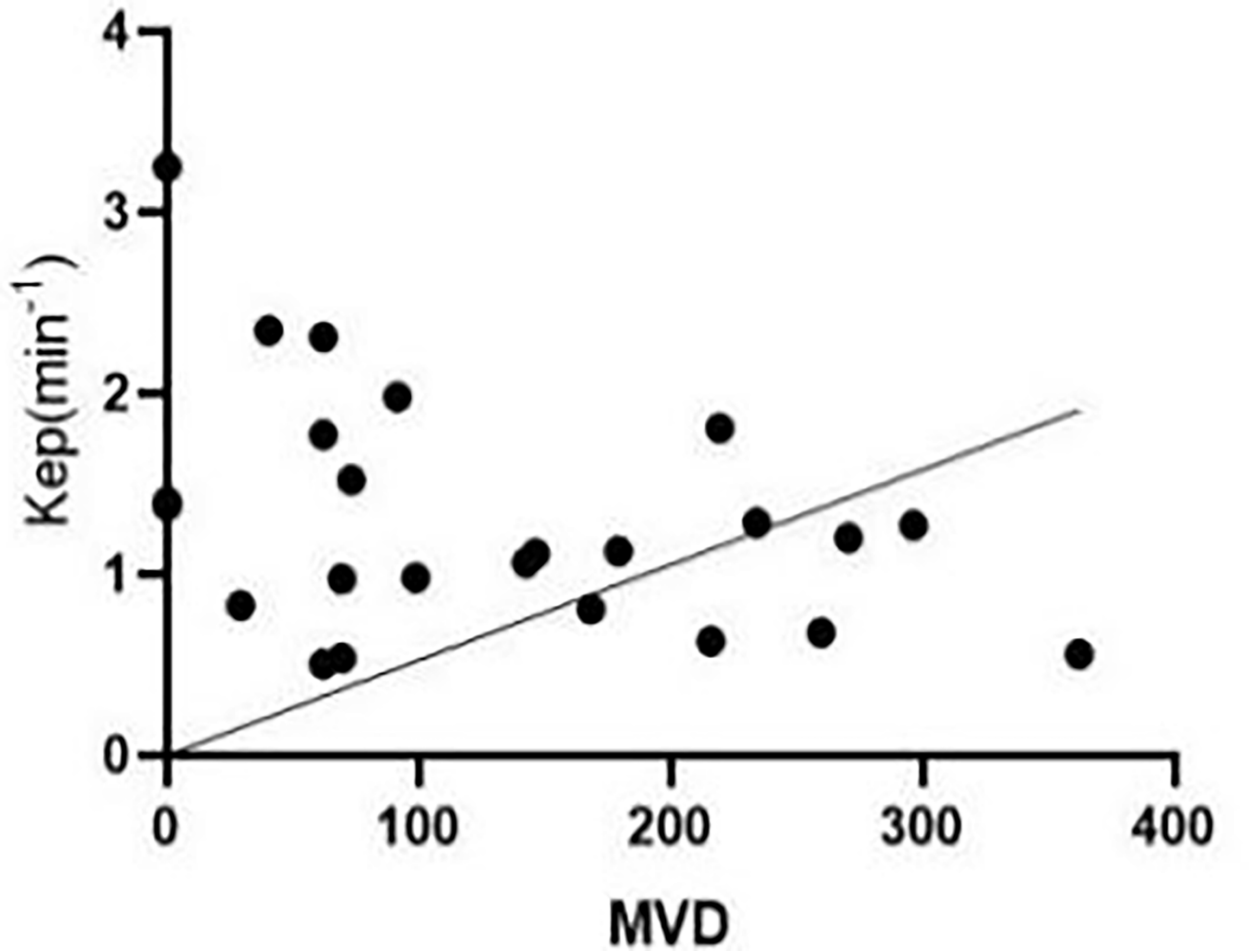
At the third week after successful modelling, microvascular density (MVD) in the peripheral blood of experimental rabbits correlated negatively with Kep.

Pearson’s correlation analysis showed that, 3 weeks after successful modelling of lower limb ischaemia, there was no correlation between peripheral blood EPCs and Kep in rabbits [r = 0.287, (95% CI: -0.131, 0.619)], while there was a negative correlation between MVD and Kep in the lateral femoral muscle of rabbits [r = -0.410, (95% CI: -0.698, -0.008)] (**Figure 6**).

**Figure 7:**
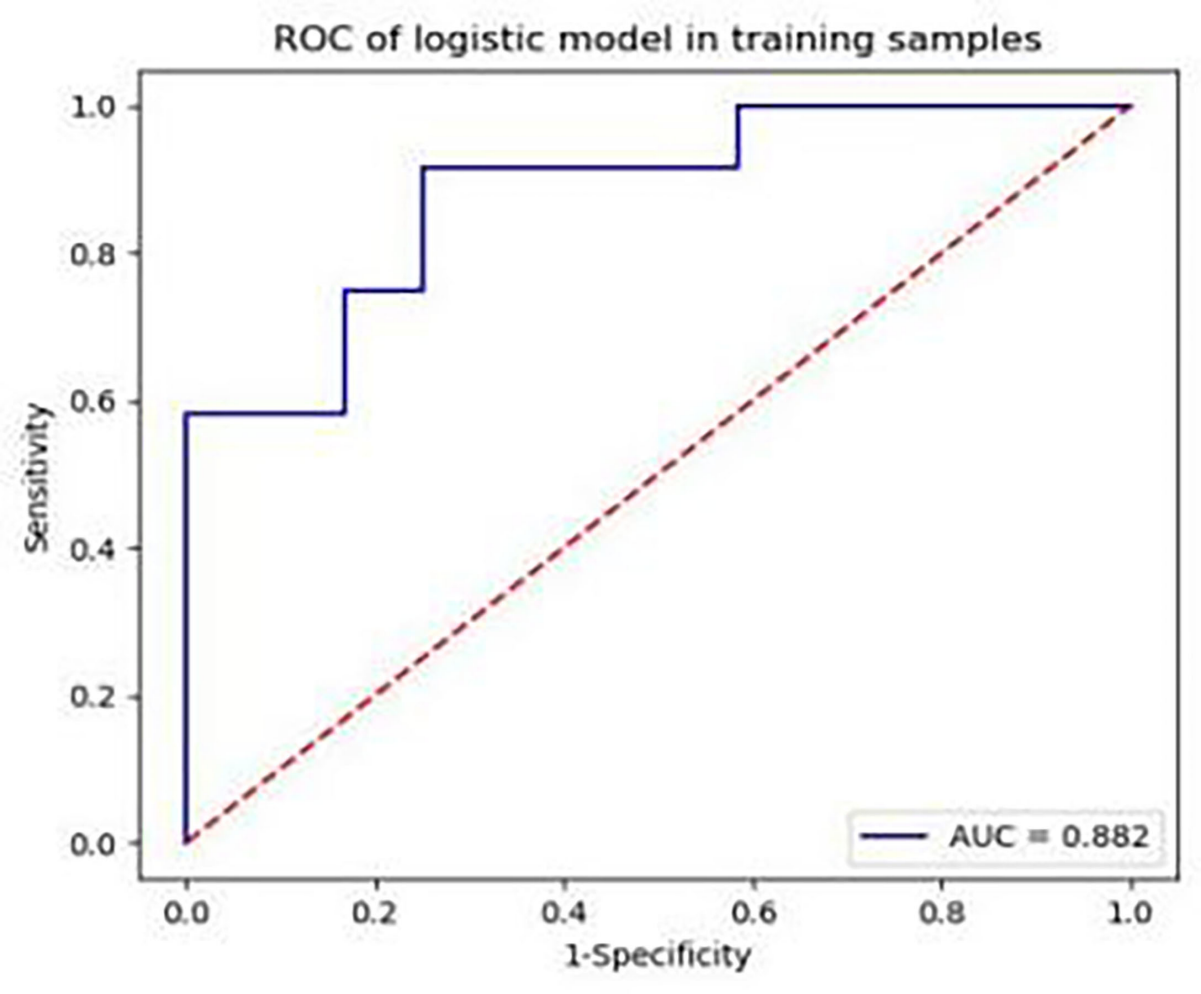
Area under the receiver operating characteristic curve of the diabetic model rabbits with critical limb ischaemia, diagnosed based on K^trans^ texture parameters, is 0.882.

From the original 63 features, 44 features were selected by *t*-test or Mann−Whitney U test, after which correlation analysis (threshold = 0.9) was performed to reduce the data redundancy. Consequently, four texture parameters were selected: MinIntensity, Quantile5, Quantile10, and Maximum 3D Diameter. MinIntensity, Quantile5, and Quantile10 are histogram parameters, and the Maximum 3D Diameter is a morphological parameter. These texture parameters were able to predict K^trans^ in the third week after establishing the CLI model, with an area under the ROC curve of 0.882 (sensitivity: 0.667, specificity: 0.833) (**Figure 7**). **Figure 7** shows the ROC area of the diabetic model with critical limb ischemia diagnosed based on Ktrans texture parameters, with a value of 0.882.

**Figure 8:**
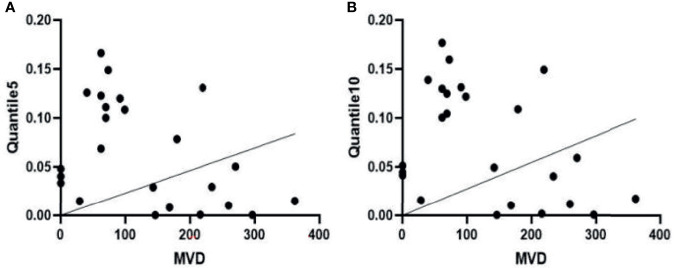
**(A)** There is a negative correlation between microvascular density (MVD) and the Quantile5 texture parameter in the femoris lateralis muscle of experimental group rabbits at week 3 after successful creation of a critical limb ischaemia model. **(B)** MVD is also negatively correlated with the texture parameter Quantile10.

**Figure 9:**
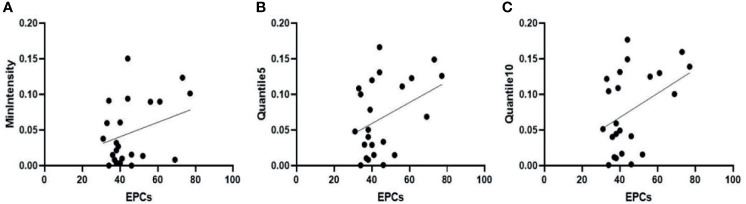
Three weeks after successfully establishing the critical limb ischaemia model in diabetic rabbits, endothelial progenitor cell counts (EPCs) are positively correlated with the texture parameters **(A)** MinIntensity, **(B)** Quantile5, and **(C)** Quantile10 of the affected limb’s lateral femoral muscle.

Pearson correlation analysis showed that MVD was negatively correlated with Quantile5 and Quantile10 [Quantile5, r = -0.418, (95% CI: -0.703, -0.018); Quantile10, r = -0.407 (95% CI: -0.696, -0.004)]. Peripheral blood EPC count positively correlated with MinIntensity, Quantile5, and Quantile10 [MinIntensity, r = 0.428 (95% CI: 0.029, 0.708); Quantile5, r = 0.463, (95% CI: 0.073, 0.730); Quantile10, r = 0.479, (95% CI: 0.094, 0.740)] (**Figures 8**, **9**). **Figures 8A, B** showed MVD was negatively correlated with the texture parameter Quantile5 and Quantile10. **Figures 9A–C** after 3 weeks of successful modeling, the experimental rabbit EPCs positively correlated with the texture parameters MinIntensity, Quantile5 and Quantile10 of the affected limb lateral femoral muscle, respectively.

## Discussion

When we investigated whether DCE-MRI texture features could be used to assess changes in muscle with CLI in a rabbit model of diabetes, we found that K^trans^ of the vastus lateralis muscle in the experimental group was decreased in the third week compared with the first week. In the third week, peripheral blood EPC counts were positively correlated with K^trans^. The texture parameters based on K^trans^, such as MinIntensity, Quantile5, and Quantile10, correlated with peripheral blood EPCs and MVD of the femoris lateralis muscle in these rabbits.

Diabetes-induced CLI is the most serious symptom of peripheral arterial disease and requires prompt treatment (7). In our study, we showed that the K^trans^ of the vastus lateralis muscle in the experimental group was decreased in the third week compared with the first week, which has not been reported previously. This is because the required microvascular perfusion of skeletal muscle cannot be fully compensated for by the collateral circulation. At the second and third weeks, K^trans^ of the experimental group was lower than that of the sham-operated group, indicating that K^trans^ can be used to evaluate skeletal muscle microvascular permeability lesions in patients with diabetes-induced CLI. This has potential guiding significance in selection of the best revascularisation strategy.

Dysfunction of bone marrow haematopoietic stem cell-derived EPCs plays a key role in diabetic vascular complications (8, 9). In this study, the number of EPCs in the experimental group was lower than that in the sham-operated group at week 3. The possible mechanism is that in the environment of hyperlipidaemia and hyperglycaemia, pro-inflammatory macrophages (M1-type) delay wound healing in diabetic patients, while anti-inflammatory macrophages (M2-type) have weakened functions that reduce VEGF production and the adhesion to endothelial cells. This results in fewer EPC migrations (10, 11). The present study showed a positive correlation between EPCs and K^trans^. Several studies (12, 13) have attempted to improve angiogenesis and increase blood flow in diabetic rats with hindlimb ischaemia by improving angiogenesis capacity and increasing the number of peripheral EPCs.

In the third week, MVD was increased in the experimental group compared with that in the sham-operated group, and collateral circulation of skeletal muscle was observed by femoral arteriography, which may be due to haem oxygenase enzyme activity increases during hypoxia, which weakens oxidative stress and inflammation. Hypoxia due to vascular endothelial cell damage can improve post-ischaemic revascularisation by activating the hypoxia-inducible factor 1-related pathway (7). K^trans^ was not correlated with MVD, because vascular endothelial cells are too insensitive to VEGF produced by hypoxia in high-glucose environments, causing a corresponding change in K^trans^. However, Kep was negatively correlated with MVD. Therefore, MVD in diabetes combined with critical lower-limb ischaemia affects the microvascular permeability of skeletal muscles. In this study, there was no significant difference in Ve between the horizontal and longitudinal studies (P > 0.05). We believe that diabetes combined with lower limb ischaemia is a chronic metabolic disease, and in this experimental animal model study, the course of disease was too short to observe significant changes. The results of our study showed that DCE-MRI microvascular permeability parameters could be used as a sensitive imaging biomarker for the severity of skeletal muscle lesions in patients with diabetes with CLI.

MRI texture analysis is a potential tool for evaluating subtle differences in the distribution patterns of muscle lesions (14–16). Our study revealed that MinIntensity, Quantile5, Quantile10, and Maximum 3D Diameter had the highest diagnostic efficacy in the model of rabbit diabetes with CLI. In the third week, the MinIntensity, Quantile5, and Quantile10 values of the experimental group were all lower than those of the sham-operated group. The Maximum 3D Diameter belongs to the morphological parameters and can be calculated according to the voxel array grey range matrix to reflect the variation in intensity or the distribution of homogeneous regions (17). The Maximum 3D Diameter increased, indicating that the skeletal muscle morphology of the K^trans^ map in the experimental group increased. The AUC value of up to 0.882 in this study suggested that texture analysis could detect changes in skeletal muscle microstructure in diabetic patients with critical lower limb ischaemia. Škochand (15) evaluated the distinction of calf muscles between individuals with and without diabetes using MR image texture features, to verify that texture analysis can be used for objective description of microstructural changes in muscles that cannot be seen by naked eye inspection. In this study, the MVD of the lateral femoral muscle was negatively correlated with the texture parameters Quantile5 and Quantile10 at the third week after model establishment. Peripheral blood EPC counts were positively correlated with MinIntensity, Quantile5, and Quantile10, suggesting that texture analysis can be used to assess the early, subtle structural changes in skeletal muscle exposed to ischaemia, There were several limitations to our study. First, the rabbit diabetic model with CLI differs from the natural course of lower limb ischaemia in patients with diabetes. Second, in this study, muscle fibre cross-sectional area and capillary number/muscle fibre number were not considered and will be studied in a later experiment.

## Conclusions

In conclusion, quantitative DCE-MRI parameters can be used to evaluate skeletal muscle microvascular permeability in diabetic rabbits with critical limb ischaemia. Texture analysis based on DCE-MRI K^trans^ is a feasible technique for evaluating the early, slight, ischaemia-related structural changes in skeletal muscle.

## Data Availability Statement

The raw data supporting the conclusions of this article will be made available by the authors, without undue reservation.

## Ethics Statement

The animal study was reviewed and approved by the Animal Experiment Center at Renmin Hospital of Wuhan University and the ethics committee at Wuhan University.

## Author Contributions

Conception and design: QY and LL. Administrative support: YFZ. Provision of study materials or patients: QY, YY, HL, DX, and YFZ. Collection and assembly of data: XD and YFZ. Data analysis and interpretation: QY, YY, HL, DX, and YFZ. Manuscript writing: HL and DX. All authors contributed to the article and approved the submitted version.

## Funding

This work was supported by the National Natural Science Foundation of China (No. 81871332), (No. 82171895).

## Conflict of Interest

The authors declare that the research was conducted in the absence of any commercial or financial relationships that could be construed as a potential conflict of interest.

## Publisher’s Note

All claims expressed in this article are solely those of the authors and do not necessarily represent those of their affiliated organizations, or those of the publisher, the editors and the reviewers. Any product that may be evaluated in this article, or claim that may be made by its manufacturer, is not guaranteed or endorsed by the publisher.

## Abbreviations

DCE, dynamic contrast agent enhanced; EPCs, endothelial progenitor cells; Kep, rate constant; K^trans,^ transfer constant; MR, magnetic resonance; MRI, magnetic resonance imaging; MVD, microvessel density; ROC, receiver operating characteristic; ROI, region of interest; Ve, volume of extravascular extracellular space.
